# Challenges faced by individuals with autism and intellectual disability as they prepare for adulthood

**DOI:** 10.1192/j.eurpsy.2025.112

**Published:** 2025-08-26

**Authors:** M. A. Ciołek

**Affiliations:** Medical University of Silesia, Katowice, Poland

## Abstract

**Abstract:**

Individuals with autism and intellectual disability encounter numerous challenges as they transition into adulthood. By analyzing case studies of students in both specialized and mainstream educational settings, I aim to highlight their perspectives and experiences.

These individuals grapple with a myriad of hurdles, from difficulties in communication and social interaction to challenges in academic and vocational pursuits. Through case studies, we witness the personal and societal barriers they encounter, highlighting the need for tailored interventions and support systems.

Early intervention and specialized education play crucial roles in empowering these individuals to thrive. By providing nurturing environments and equipping them with essential skills, we can foster independence and enhance their overall well-being. Additionally, community resources offer ongoing support beyond the school setting, further facilitating their transition into adulthood.

Central to our discussion is the importance of fostering an inclusive society that embraces neurodiversity. When individuals with autism and intellectual disability are valued for their unique perspectives and talents, they can contribute meaningfully to society. Through acceptance and understanding, we can pave the way for a more equitable future for all.

**Disclosure of Interest:**

None Declared

